# SEL1L: molecular gatekeeper of the endoplasmic reticulum quality control network

**DOI:** 10.3389/ebm.2026.11350

**Published:** 2026-10-30

**Authors:** Zhenxuan Shen, Yiting Niu, Hedong Chen, Meikun Pan, Fan Yang, Meiying Li, Chong Zhang, Yali Zhou, Jianguo Yan

**Affiliations:** 1 Guangxi Key Laboratory of Brain and Cognitive Neuroscience, Guilin Medical University, Guilin, Guangxi, China; 2 Department of Neurology, The Second Affiliated Hospital of Guilin Medical University, Guilin, China; 3 Guangxi Medical and Health Key Cultivation Discipline Construction Project, Guilin, Guangxi, China; 4 Institute of Pathogenic Biology, Guilin Medical University, Guilin, Guangxi, China

**Keywords:** AD, immunity, metabolic diseases, neurodegenerative diseases, PD, SEL1L, tumor

## Abstract

SEL1L (suppressor/enhancer of lin-12-like) is an endoplasmic reticulum (ER) transmembrane protein that serves as a key regulatory molecule in the endoplasmic reticulum-associated degradation (ERAD) pathway, playing a central role in maintaining intracellular protein homeostasis. This paper provides a systematic review of SEL1L’s structural characteristics, the molecular mechanisms by which it forms a functional complex with the E3 ubiquitin ligase HMG-CoA reductase degradation protein 1 (HRD1, encoded by *SYVN1*) to synergistically execute ERAD, and its pathophysiological significance in the onset and progression of various diseases. Research indicates that SEL1L dysfunction is closely associated with pathogenic protein aggregation in neurodegenerative diseases (e.g., Alzheimer’s disease, Parkinson’s disease, amyotrophic lateral sclerosis), protein homeostasis imbalances during tumorigenesis, cellular metabolic dysregulation in metabolic disorders (e.g., diabetes, lipid metabolism disorders), and immune system dysfunction. Furthermore, the expression levels or variants of SEL1L exhibit specific alterations across different tumor tissues, suggesting its potential as a disease biomarker and therapeutic target. This paper aims to synthesize existing research, elucidate the ‘gatekeeper’ role of SEL1L in multiple physiological and pathological processes, and explore the prospects for developing novel therapeutic strategies by targeting the SEL1L-HRD1 complex and related pathways.

## Impact statement

The endoplasmic reticulum-associated degradation pathway is essential for cellular protein homeostasis. While recent reviews examine SEL1L within narrow contexts like cancer or neurodegeneration, a systematic synthesis of its roles across diverse disease groups is still missing. This review creates a unified framework covering SEL1L’s structural biology, molecular mechanisms, and broad functions in neurodegeneration, cancer, metabolic disease, immunity, and viral infection. It consolidates recent progress, including tissue-specific protein substrates and newly identified links to viral infection, establishing SEL1L as a central hub connecting endoplasmic reticulum quality control to widespread pathological processes. We highlight the largely untapped therapeutic potential of targeting the SEL1L-HRD1 complex. This synthesis defines key knowledge gaps and offers fresh perspectives for developing biomarker-guided, mechanism-based therapeutic strategies across multiple human diseases.

## Introduction

SEL1L (suppressor/enhancer of lin-12-like) has emerged as a significant focus of biomedical research, with its structure and function attracting considerable attention. The SEL1L gene was first isolated from human chromosomes in 1997 by Biunno et al. Sequence analysis revealed significant homology between human SEL1L and the *Sel-1*gene of *Caenorhabditis elegans*, suggesting that SEL1L belongs to an evolutionarily conserved family of ER protein quality-control factors [1]. Subsequent structural and biochemical studies have shown that SEL1L is an ER-resident membrane protein containing conserved Sel1-like repeat (SLR) motifs in its luminal region, which contribute to SEL1L oligomerization and binding to HRD1 [2], whereas its transmembrane domain mediates association with HRD1 and regulates ERAD-L [3]. Notably, as a key regulatory component of the ERAD pathway, SEL1L plays an indispensable role in maintaining intracellular protein homeostasis. Recent studies have linked SEL1L or the SEL1L–HRD1 ERAD pathway to tumor biology, immune regulation, and neurological disorders: SEL1L expression correlates with glioma progression and poor clinical outcome [4], the SEL1L–HRD1 complex limits STING-mediated innate immune signaling by targeting nascent STING for degradation [5], and biallelic hypomorphic variants affecting SEL1L–HRD1 ERAD are associated with neurodevelopmental disorders [6]. Its aberrant expression or functional deficiency is closely associated with multiple pathological processes. Recent identification of disease-causing germline variants in SEL1L and HRD1 has provided direct human genetic evidence for the essential role of SEL1L-HRD1 ERAD in neurodevelopment, immune function, and cellular proteostasis. These findings further support the concept that SEL1L functions as a tissue-sensitive gatekeeper of ER homeostasis and cell fate. This review summarizes the latest research on the SEL1L molecule, explores its mechanisms of action in different diseases, and prospects its future applications, aiming to provide reference for researchers in related fields.

From an evolutionary perspective, the Hrd3p–Hrd1p/SEL1L–HRD1 adaptor–E3 axis represents a conserved ERAD branch from yeast to mammals, although its nomenclature and accessory components differ among species. In *Saccharomyces cerevisiae*, Hrd3p contributes to Hrd1p substrate selection and Cdc48p recruitment [7]. Structural studies characterized the Hrd1p–Hrd3p complex [8] and showed that Hrd3p/Yos9p recognize glycosylated luminal substrates, whereas Hrd1p/Der1p mediate retrotranslocation [9]. In *Caenorhabditis elegans*, HRD-1/SEL-11 participates in ERAD, the unfolded protein response, and developmental growth [10, 11]. In mammals, SEL1L is a core HRD1 adaptor required for functional complex assembly and activity [12, 13], making the SEL1L–HRD1 complex a functional counterpart, rather than an identical orthologue, of the yeast Hrd3p–Hrd1p module. SEL1L-dependent ER functions regulate LPL secretion independently of Hrd1-mediated ERAD [14], whereas SEL1L–HRD1 ERAD regulates systemic metabolism through the CREBH–FGF21 axis [15], limits basal STING signaling [5], and suppresses hepatocyte hyperproliferation and liver tumorigenesis [16]. Collectively, these findings support a gatekeeping role for SEL1L–HRD1 in tissue-specific ER proteostasis and downstream cellular functions.

## SEL1L molecule

### Introduction to SEL1L molecules

The SEL1L molecule is encoded by the SEL1L gene (suppressor/enhancer of lin-12-like), which maps to human chromosome 14q24.3-q31 and encodes an ER-resident transmembrane protein. The SEL1L gene contains 21 exons and generates multiple alternatively spliced transcripts encoding distinct protein isoforms. The full-length SEL1L protein consists of four major segments: 1. An N-terminal signal peptide (15–30 hydrophobic amino acids) responsible for ER targeting; 2. A large luminal region containing an N-terminal fibronectin type II (FNII) domain followed by tandem SEL1L-like repeats (SLRs). These SLRs adopt conserved α-helical and β-sheet secondary structures to constitute the primary substrate-binding interface, with the C-terminal SLR segment required for SEL1L–HRD1 interaction and formation of the functional HRD1 ERAD complex; 3. A single transmembrane α-helix (∼20–25 hydrophobic residues) located C-terminally to the SLR region; 4. A short C-terminal cytoplasmic tail oriented toward the cytosol, consisting of approximately 24–35 amino acids with a proline-rich sequence. To date, there is no experimental evidence confirming verified functional phosphorylation sites within this cytoplasmic tail or phosphorylation-dependent regulation of SEL1L by cellular signaling pathways. Alternative splicing of SEL1L transcripts enables isoform diversity and supports substrate recognition specificity during ER-associated degradation [13], as illustrated in Figure 1.

**FIGURE 1:**
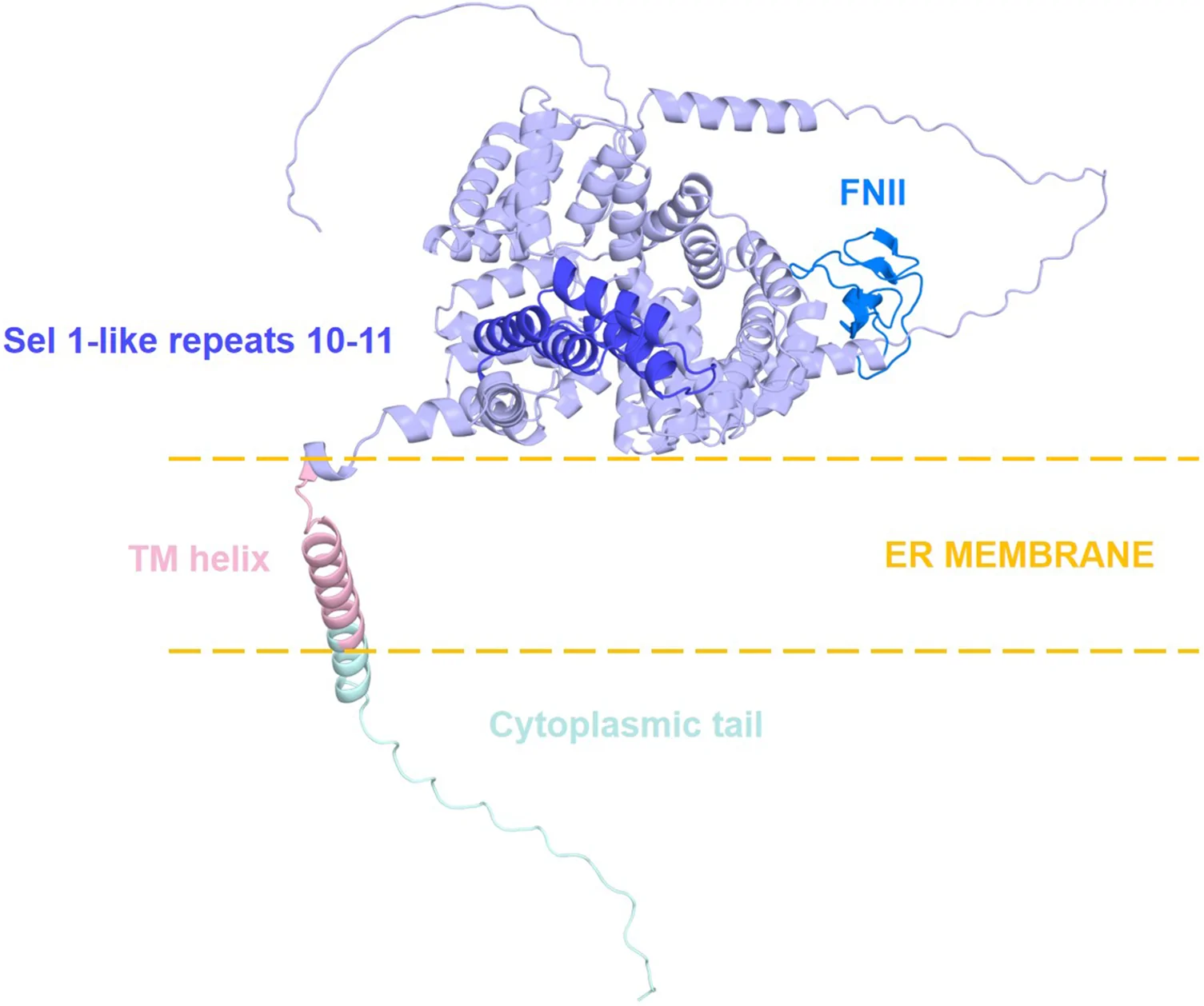
Structural organization of SEL1L. SEL1L is an ER-resident transmembrane protein with a large ER-luminal region containing the fibronectin type II (FNII) domain and Sel1-like repeats (SLRs; SLR10–11 highlighted), followed by a transmembrane (TM) helix and a short cytoplasmic tail. The dashed lines indicate the ER membrane. The structural features are illustrated based on previous structural studies [2, 3].

### Endoplasmic reticulum-associated degradation

The ER is a vital organelle in eukaryotic cells, serving as the starting point and central hub of the protein secretion pathway. It performs multiple critical functions, including the synthesis and transport of secretory and membrane proteins, as well as the synthesis of lipids. These proteins must undergo proper folding and packaging; otherwise, misfolded or unfolded proteins will become trapped within the ER and exhibit certain toxicities. To ensure the proper folding and production of thousands of proteins within the ER while promptly eliminating misfolded ones, eukaryotic cells have evolved a complex quality control mechanism capable of removing terminally misfolded or unassembled peptides [17]. This mechanism is termed ERAD. The discovery of ERAD dates back to the late 1980s, when multiple research groups reported that misfolded peptides or unassembled protein complex subunits often remain trapped in the ER. However, they are recognized by ERAD and retrotransported to the cytoplasm for rapid degradation via ubiquitination. This ubiquitination-mediated degradation process is primarily carried out by the ubiquitin-proteasome system (UPS). The UPS is a degradation mechanism operating in the cytoplasm and nucleus, whereas ERAD is a specific pathway tailored for the unique structure of the ER. Thus, ERAD can be defined as a specialized protein quality control mechanism dependent on the UPS [18], representing a specialized branch of the UPS within the ER. ERAD also responds to metabolic disturbances by regulating the levels of enzymes or signaling molecules [14, 15].

ERAD is a multi-step process: the first step involves the recognition of damaged proteins, followed by their retrotransport from the ER to the cytoplasmic matrix and subsequent degradation in the cytoplasm [17]. This process encompasses multiple coordinated and substrate-specific steps, including substrate recognition, processing, retrotranslocation, ubiquitination, and proteasomal degradation [17]. In mammalian cells, ER-luminal chaperones and lectins, including BiP, EDEM1–3, OS-9/XTP3-B, and ERdj5, retain, remodel, or deliver misfolded proteins to downstream ERAD machinery; ERdj5 can cooperate with BiP to reduce disulfide-linked ERAD substrates [17, 19]. The selected substrates are subsequently delivered to ER membrane-associated retrotranslocation modules. In mammals, the SEL1L–HRD1 complex represents a major ERAD-L pathway, whereas Derlin-1-containing complexes cooperate with p97/VCP in the retrotranslocation of selected substrates [20, 21]. By contrast, Der1p and Dfm1p are yeast ERAD components; the yeast Hrd1p complex contains Hrd1p, Hrd3p, Yos9p, Der1p, and Usa1p [9], whereas Dfm1p contributes to the retrotranslocation of integral membrane ERAD substrates [22]. Substrate ubiquitination may occur before or during retrotranslocation, followed by p97/VCP-assisted extraction and proteasomal degradation [9, 17, 20–22]. Ubiquitin is a highly conserved protein composed of 76 amino acid residues, widely present in eukaryotic cells either as monomers or in complexes with other proteins. The process of covalently attaching degradation signals to ubiquitin molecules on proteins destined for degradation is called ubiquitination. The vast majority of ubiquitinated proteins are tagged for degradation. Finally, in the third step, ubiquitinated proteins are degraded by relevant proteasomes to relieve endoplasmic reticulum stress (ERS), preventing ER dysfunction and subsequent apoptosis that could lead to pathological conditions.

The SEL1L molecule also functions as an ER transmembrane linker protein for HRD1, participating in ER metabolism and quality control [3, 20]. HRD1 is an E3 ubiquitin ligase [23]. In the protein ubiquitination pathway, ubiquitin is activated by E1 activators, transferred to conjugating enzyme E2 to form a ubiquitin-conjugating enzyme complex, and then transferred from E2 to the target protein with the participation of E3 ubiquitin ligases. Different ubiquitin molecules link together to form short polymer chains. Ubiquitinated target proteins are recognized by the 26S proteasome and hydrolyzed into small fragments [3, 20]. In this process, E3 ubiquitin ligases primarily mediate the attachment of ubiquitin molecules to target proteins for labeling. Subsequently, with the assistance of ATPases, misfolded or unfolded proteins are transported to the cytoplasm and ultimately degraded by associated proteins. The specificity of this ubiquitination depends on the type of linkage formed between ubiquitin molecules. Thus, the E3 ubiquitin ligase responsible for this linkage plays a crucial role in degrading target proteins. HRD1 is the key E3 ubiquitin ligase in ERAD, while SEL1L primarily recruits and stabilizes HRD1 within ERAD.

### SEL1L–HRD1 within the broader ER quality-control network

ERAD represents one branch of the broader endoplasmic reticulum protein quality-control network, which integrates luminal chaperones and lectins, ER membrane-associated retrotranslocation factors, cytosolic p97/VCP extraction machinery, ubiquitin-conjugating enzymes, and the 26S proteasome [18, 24]. Numerous ER-localized E3 ubiquitin ligases have been identified and form partially overlapping but functionally distinct ERAD branches [25]. Representative examples include HRD1/SYVN1, AMFR/gp78, MARCHF6/TEB4, RNF5/RMA1, RNF139/TRC8, and TMEM129. Among these, TMEM129 has been identified as an ERAD E3 ligase involved in the degradation of specific membrane substrates [26]. These ligases mediate the ubiquitination and clearance of misfolded luminal and membrane proteins and also connect ER quality control with lipid metabolism, innate immune signaling, antiviral defense, and tumor-associated processes. For example, AMFR/gp78 participates in sterol-regulated degradation of HMG-CoA reductase and has been implicated in tumor progression and protein-misfolding disorders [27, 28]. MARCHF6 regulates cholesterol synthesis by promoting the degradation of multiple sterol-biosynthetic enzymes [29]. RNF5 modulates ER-stress-associated signaling in acute lung injury and also influences antitumor immunity and intestinal inflammation [30–32]. TMEM129 represents a distinct branch of the Derlin-1-associated ERAD pathway involved in the degradation of membrane proteins [26]. Within this network, HRD1 acts as a major ERAD E3 ubiquitin ligase, whereas SEL1L functions as an essential ER-resident adaptor that stabilizes HRD1 and couples substrate recognition to retrotranslocation and ubiquitination [13, 20, 23]. Thus, SEL1L–HRD1 shares with other ER E3 ligases the common functions of substrate ubiquitination, extraction, and proteasomal degradation, but is distinguished by its obligate adaptor–E3 architecture, broad tissue-specific substrate repertoire, and direct human genetic evidence linking SEL1L–HRD1 dysfunction to neurodevelopmental disease, agammaglobulinemia, and immunological abnormalities [6, 33], as illustrated in Figure 2.

**FIGURE 2:**
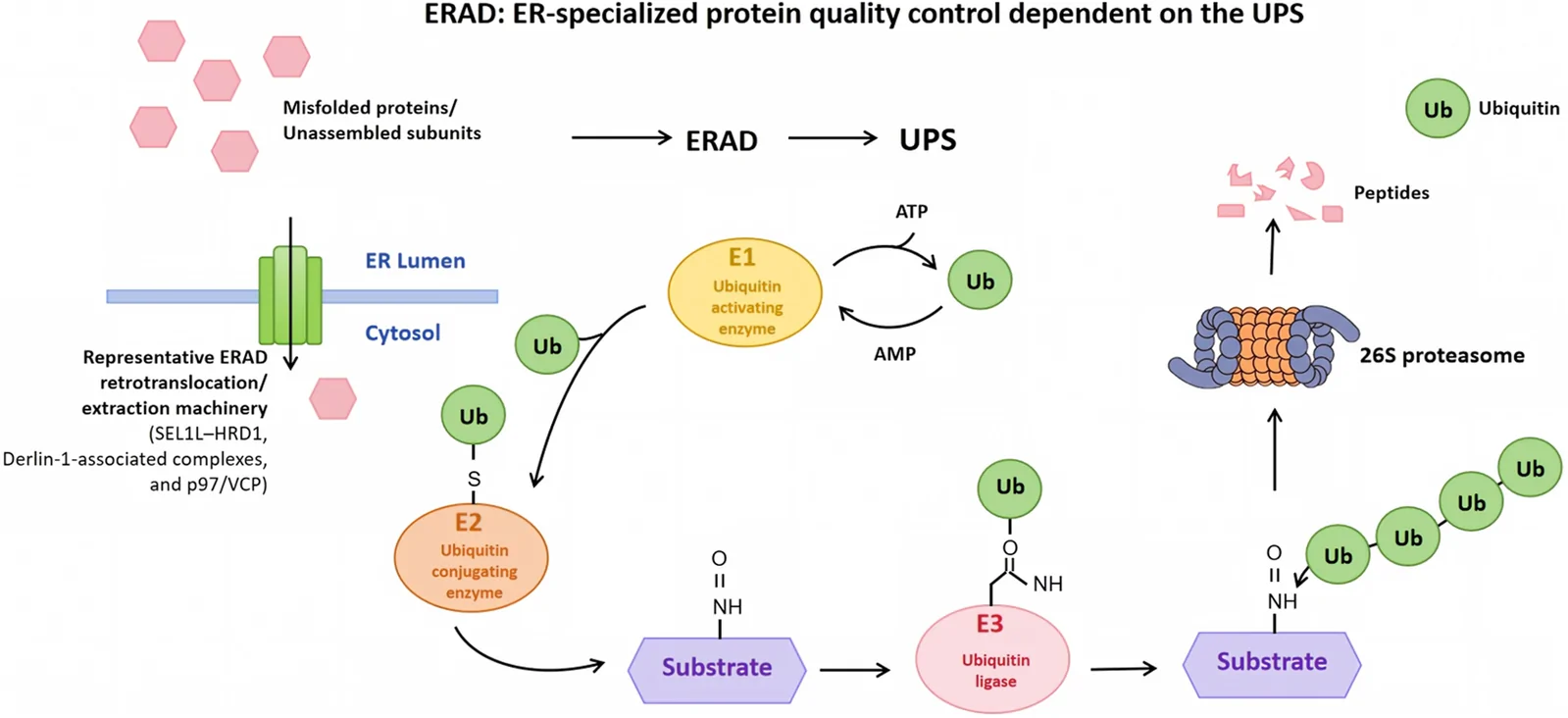
Schematic overview of ER-associated degradation (ERAD) and its coupling to the ubiquitin–proteasome system (UPS). Misfolded ER proteins are recognized by ER quality-control factors, retrotranslocated through representative ERAD membrane modules, extracted by cytosolic p97/VCP, ubiquitinated through the E1–E2–E3 cascade, and ultimately degraded by the 26S proteasome. For simplicity, SEL1L–HRD1, Derlin-1-associated complexes, and p97/VCP are grouped as representative ERAD retrotranslocation/extraction machinery in the schematic and do not indicate a single stable complex.

ERAD is a relatively conserved ER quality control mechanism in cells [34]. SEL1L, an adaptor protein on the ER membrane, serves as a key factor in the ERAD mechanism and is an indispensable linker in the HRD1 pathway among several retrotranslocation pathways. SEL1L stabilizes and recruits HRD1 to participate in ERAD. SEL1L recognizes and pairs with misfolded or unfolded substrates from ERAD. Within ERAD, it primarily ensures the accurate delivery of abnormal proteins into the HRD1 transport channel. HRD1 then attaches ubiquitin molecules to the target proteins for labeling, after which a series of cytoplasmic ATPases and the 26S proteasome degrade the marked proteins, as illustrated in Figure 3.

**FIGURE 3:**
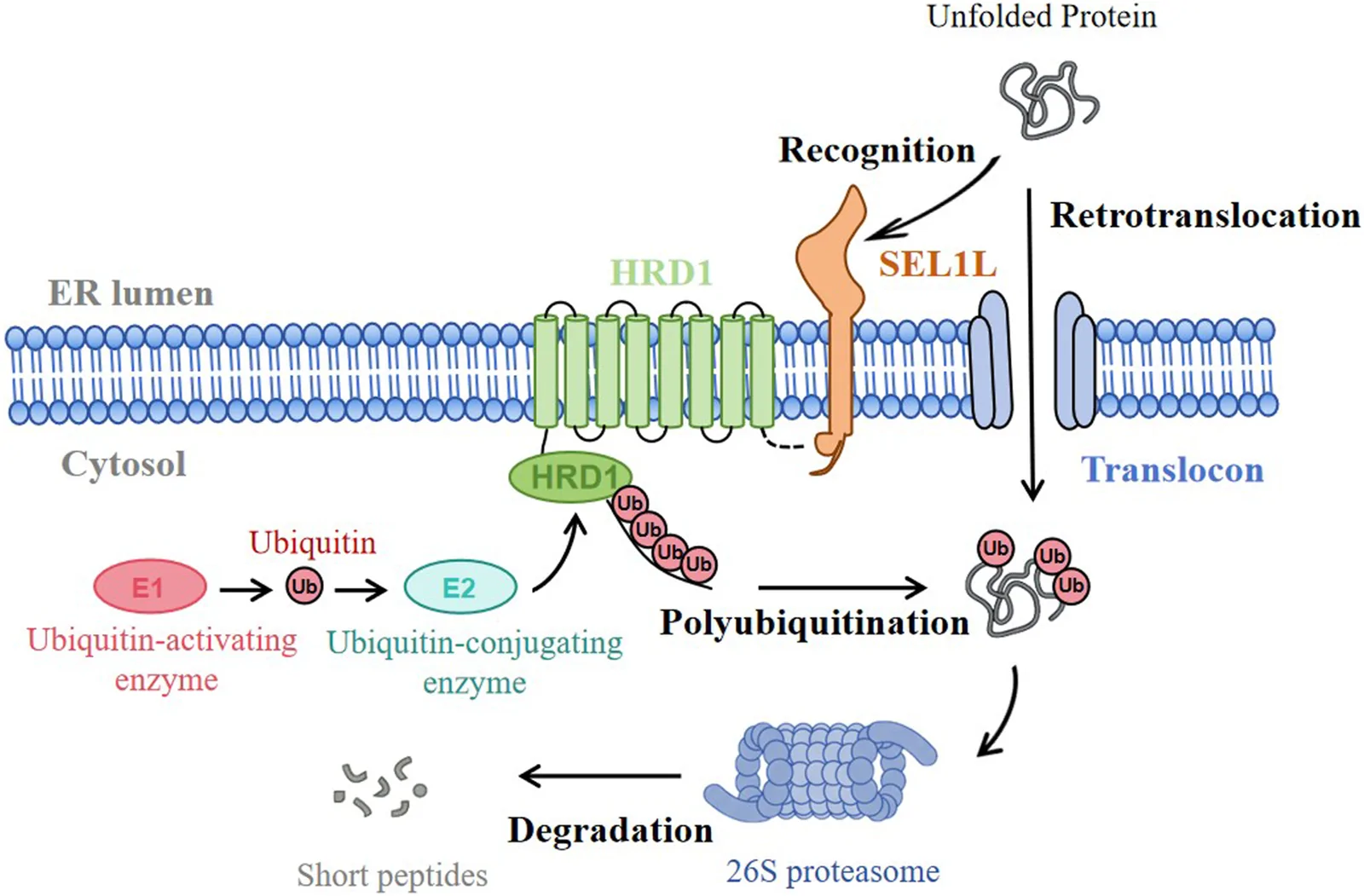
Schematic representation of the mammalian SEL1L–HRD1 ERAD pathway. HRD1 is shown as an ER membrane E3 ubiquitin ligase containing eight transmembrane helices, whereas SEL1L is shown as a single-pass ER membrane adaptor. Misfolded ER proteins are recognized and transported across the ER membrane, ubiquitinated through the E1–E2–HRD1 cascade, and subsequently degraded by the 26S proteasome. This figure was newly drawn as an original schematic by the authors based on reported studies of the mammalian SEL1L–HRD1 complex [12, 13, 20].

Studies have demonstrated that SEL1L exhibits strong correlation and interaction with HRD1 during the ERAD process. SEL1L serves as the transmembrane adaptor protein for HRD1. Primarily participates in ERAD by recruiting, stabilizing, and binding to HRD1 to form the SEL1L-HRD1 complex [15]. Proteomics screening experiments involving SEL1L and HRD1 demonstrate that their interaction forms a functional ERAD complex, contributing to ERAD by recruiting substrates, facilitating their retrotranslocation, and promoting their ubiquitination [35]. Research indicates that the SEL1L molecule is indispensable for maintaining HRD1 stability, normal ERAD function, and ER homeostasis. Experimental SEL1L deficiency destabilizes HRD1 and impairs SEL1L–HRD1 ERAD, leading to ER stress, misfolded-protein accumulation, ER abnormalities, and cell-type-specific tissue pathology. These effects vary according to the affected cell type, tissue, and experimental model. To date, the strongest direct human genetic evidence involves biallelic hypomorphic variants rather than complete loss-of-function alleles.

### Germline SEL1L–HRD1 variants and human disease

Experimental studies have established the physiological importance of SEL1L–HRD1 ERAD, while germline variants provide direct evidence linking impaired ERAD to human disease. The best-characterized cases are biallelic hypomorphic variants in SEL1L or HRD1 associated with infantile-onset ERAD-associated neurodevelopmental disorder (ENDI).

Wang et al. identified three biallelic missense variants in six children from three unrelated families. The patients presented with developmental delay, intellectual disability, microcephaly, facial dysmorphism, hypotonia, and/or ataxia. SEL1L p.Gly585Asp impaired substrate recruitment, SEL1L p.Met528Arg disrupted SEL1L–HRD1 complex formation and stability, and HRD1 p.Pro398Leu reduced HRD1 activity. These findings demonstrated that partial impairment of SEL1L–HRD1 ERAD causes human neurodevelopmental disease [6].

A more severe phenotype was reported in five patients from two consanguineous Slovakian families carrying the biallelic SEL1L p.Cys141Tyr variant. The patients developed early-onset neurodevelopmental abnormalities, agammaglobulinemia, recurrent infections, and premature death. This variant disrupts a disulfide bond in the SEL1L luminal FNII domain and severely impairs SEL1L–HRD1 ERAD through mutant-protein aggregation and HRD1-mediated degradation of SEL1L. The condition was designated ENDI with agammaglobulinemia (ENDI-A), and its severity is consistent with an inverse relationship between residual ERAD activity and disease severity [33].

Disease-associated SEL1L variation was also identified in animals before the discovery of human ENDI. A homozygous SEL1L p.Ser658Pro variant caused early-onset progressive cerebellar ataxia, Purkinje-cell loss, and cerebellar ER-stress responses in Finnish Hounds [36]. Similarly, Purkinje cell-specific SEL1L deletion caused progressive cerebellar ataxia, ER dilation, and Purkinje-cell loss in mice [37].

Together, these findings support a model in which disease-associated variants disrupt multiple levels of SEL1L–HRD1 ERAD, including substrate recruitment, complex assembly, HRD1 activity, and SEL1L stability. Residual ERAD activity may therefore influence tissue-specific proteostasis and disease severity. Clinically unaffected heterozygous relatives are consistent with autosomal-recessive inheritance and suggest that one functional allele may be sufficient to prevent an overt phenotype [6, 33]. Reported manifestations include neurodevelopmental abnormalities, immunodeficiency, impaired B-cell maturation, recurrent infections, and premature death.

A recent study explored an ASO-based strategy for the SEL1L p.Cys141Tyr defect. In knock-in mice and patient-derived fibroblasts, exon skipping generated a truncated but functional SEL1L isoform, partially restoring ERAD activity and rescuing developmental and B-cell abnormalities. These findings provide preclinical proof-of-concept for RNA-splicing modulation in SEL1L-related ERAD deficiency [38].

Currently, the best-established human disease-associated variants are biallelic missense changes in SEL1L or HRD1. The contribution of loss-of-function, splice-altering, or structural variants remains unclear. Further genotype–phenotype studies are needed to determine how variant position, residual ERAD activity, and tissue-specific substrate dependence influence clinical severity. Consistent with this model, SEL1L–HRD1 interface variants in knock-in mice produced graded phenotypes, ranging from complete neonatal lethality to partial lethality or normal viability, depending on the extent of complex disruption [39]. Neurodevelopmental abnormalities are the most consistent clinical manifestation reported to date, although the limited number of cases prevents definitive conclusions regarding tissue-specific vulnerability.

## SEL1L plays an indispensable role in neurodegenerative diseases

Neurodegenerative diseases (NDs) constitute a class of disorders primarily characterized by neuronal damage in specific regions of the nervous system and degenerative changes in nerve cells and/or their myelin sheaths. Unlike other diseases, NDs are marked by the progressive loss of selectively vulnerable neuronal populations. The emergence of protein aggregates serves as a hallmark and common feature across these conditions, with common examples including amyloid beta proteins, tau proteins, α-synuclein, and TDP-43. These aggregates often bind to ubiquitin-proteasome system subunits and certain molecular chaperones [40]. This indicates that impairment of the pathogenic protein degradation mechanism is the key cause of disease onset, providing a major research direction for scholars. Progressive neuronal dysfunction and death primarily cause cognitive and behavioral impairments, which are the most common clinical manifestations [41]. As neurodegenerative diseases involve multiple cells and systems [42], their high incidence, treatment challenges, limited therapeutic options, and poor outcomes make them one of the incurable diseases threatening human health. Nevertheless, since the discovery of neurodegenerative diseases, humanity has relentlessly pursued their underlying mechanisms and therapeutic approaches. Current treatments can improve patients’ quality of life; for example, in Parkinson’s disease, levodopa and deep brain stimulation alleviate tremors [42].

As the neurodegenerative diseases with the highest global prevalence [43, 44], Parkinson’s disease (PD) and Alzheimer’s disease (AD) severely impact patients’ daily lives, imposing a heavy burden on individuals, families, and society. Other NDs include Huntington’s disease (HD), amyotrophic lateral sclerosis (ALS), Kennedy disease (KD), Familial Amyloidotic Polyneuropathy (FAP), Age-related Macular Degeneration (AMD), and so on. The pathogenesis of neurodegenerative diseases remains unclear, but ERS, oxidative stress, protein toxicity stress, and associated abnormalities in the ubiquitin-proteasome and autophagy/lysosomal systems, neuroinflammation, and genetic factors are all closely implicated [42]. As previously noted, SEL1L occupies an indispensable position within the ER and is closely associated with ERAD. Research indicates that the primary pathological changes in PD involve significant degeneration and loss of dopaminergic neurons in the substantia nigra of the midbrain, alongside the appearance of Lewy bodies composed primarily of misfolded α-synuclein [45]; AD arises from mutations in the presenilin 1/2 (PSEN1/2) genes, the amyloid precursor protein (APP) gene, and its apolipoprotein E (APOE) gene. Its crucial pathological changes involve initial or primary alterations in amyloid metabolism, leading to the deposition of β-amyloid (Aβ) and tau protein [46, 47].

### SEL1L and PD

Parkinson’s disease, often termed “paralysis agitans,” is the world’s second most prevalent neurodegenerative disorder [44], first described by British physician James Parkinson in 1817. Its primary cause is the degeneration and death of dopaminergic neurons in the substantia nigra of the midbrain, leading to a marked reduction in striatal dopamine levels. This condition is associated with multiple factors including genetics, environmental influences, and neurological aging [48]. Symptoms vary widely but fall into two main categories: motor and non-motor [49]. Motor symptoms include resting tremor, muscle rigidity, bradykinesia, and balance disorders. Non-motor symptoms primarily encompass constipation, hyposmia, sleep disturbances, autonomic dysfunction, and psychiatric/cognitive impairments.

PD is also characterized by the accumulation of misfolded α-synuclein in Lewy bodies [50]. ER stress and impaired ER protein-quality-control mechanisms have been implicated in PD pathogenesis. HRD1 has been shown to promote the ubiquitination and degradation of Pael-R, a substrate of Parkin whose accumulation is associated with dopaminergic neuronal toxicity [51]. HRD1-mediated degradation of amyloid precursor protein (APP) has also been demonstrated, and loss of HRD1-mediated degradation causes APP accumulation and increased amyloid-β generation [52]. These findings establish Pael-R and APP as experimentally validated HRD1 substrates; however, they do not directly demonstrate that their degradation is mediated by the complete SEL1L–HRD1 complex.

SEL1L may contribute to neuronal protection by maintaining HRD1 stability and ERAD function. In a 6-hydroxydopamine-induced cellular model of PD, suppression of SEL1L enhanced neuronal cell death and was accompanied by reduced HRD1 protein levels [53]. In addition, inhibition of miR-101 protected SH-SY5Y cells against 6-hydroxydopamine-induced cell death by preserving SEL1L expression [54]. Although α-synuclein misfolding and impaired protein homeostasis are important features of PD [55], increased Sel1l expression reported in α-synuclein transgenic mice provides only associative evidence and does not demonstrate direct α-synuclein degradation by SEL1L–HRD1 [56]. Accordingly, α-synuclein should be regarded as a candidate or indirectly affected protein rather than a confirmed SEL1L–HRD1 substrate. Similarly, amyloid-β is a downstream product of APP processing and should not be described as an independently validated SEL1L–HRD1 ERAD substrate. Current evidence therefore supports the involvement of HRD1/SEL1L-associated ER proteostasis in PD, particularly through the regulation of Pael-R and neuronal stress responses, whereas the specific role of SEL1L–HRD1 in α-synuclein proteostasis remains to be clarified.

### SEL1L and AD

Alzheimer’s disease, as the most prevalent neurodegenerative disorder globally, is projected to triple its patient population worldwide by 2050, with biological definitions indicating a prevalence three times higher than clinical diagnoses. This staggering projection underscores the urgent need for prevention and treatment strategies. However, numerous factors may contribute to the onset and progression of AD. Mitochondrial dysfunction, oxidative stress, selective autophagy, vascular abnormalities, neuroinflammation, and aging are currently recognized as significant influencing factors [46]. Substantial evidence indicates genetic factors play a role in AD pathogenesis, with mutations in the apolipoprotein E (ApoE) gene and the ε4 allele being particularly significant, associated with a 3-4 fold increase in AD risk. Additionally, mutations in the amyloid precursor protein gene (APP), presenilin 1 gene (PSEN1), and presenilin 2 gene (PSEN2) are closely associated with AD onset, particularly in patients with early-onset disease manifesting clinical symptoms before age 65. The primary pathological hallmarks of AD are extracellular deposits of β-amyloid protein (Aβ) and the formation of neurofibrillary tangles. The former manifests as amyloid plaques, also known as “senile plaques,” while the latter represents intracellular deposits resulting from excessive phosphorylation of tau protein [57]. Numerous studies indicate that Aβ accumulation contributes to neuronal injury in AD. APP is synthesized and processed through the ER–Golgi secretory pathway, and γ-secretase cleavage generates the aggregation-prone Aβ40 and Aβ42 peptides [57]. Mechanistic studies have shown that HtrA2/Omi binds immature APP at the cytosolic face of the ER and cooperates with the proteasome in ER-associated degradation. Inhibition or loss of HtrA2-mediated ERAD stabilizes APP, increases APP C-terminal fragments, and enhances Aβ secretion [58]. In the hippocampus of patients with AD, hHrd1 immunoreactivity is increased in selected neuronal and astrocytic populations and is associated with hyperphosphorylated tau [59]. In addition, increased active HtrA2/Omi in the AD brain has been reported to correlate with cholinergic alterations [60]. However, the available studies do not directly demonstrate altered SEL1L mRNA expression in AD. Therefore, current evidence supports a possible involvement of ERAD- and HRD1-associated proteostasis mechanisms in AD, whereas the specific role of SEL1L in AD remains to be clarified.

### SEL1L and amyotrophic lateral sclerosis (ALS)

Amyotrophic lateral sclerosis (ALS), first described by French neurologist Jean-Martin Charcot, is the most common motor neuron disease in adults. It is a highly lethal neurological disorder characterized by degeneration of upper motor neurons (UMNs) and lower motor neurons (LMNs) in the spinal cord regions. Clinically, ALS exhibits significant heterogeneity, primarily determined by the balance between UMN and LMN degeneration [61], leading to variations even within individual cases [62]. However, it is typically characterized by muscle spasticity and atrophy, progressive skeletal muscle weakness, bulbar palsy, and pyramidal tract signs [63]. Over half of ALS patients exhibit damage to the frontal-temporal lobes of the brain, with some developing frontotemporal dementia (FTLD). Approximately 10% of cases occur in familial ALS, suggesting a genetic link [61]. It is now widely accepted that SOD1 gene mutations are closely linked to ALS pathogenesis. SOD1 mutations cause the SOD1 protein to become conformationally unstable, forming insoluble toxic protein aggregates [64]. These aggregates may exert toxic effects on motor neurons controlling muscles. SOD1 interacts with Derlin-1 in the ERAD mechanism, triggering ERS and ultimately leading to neuronal death. Research demonstrates that the IRE1α-XBP1 and ATF6 pathways within the ER unfolded protein response (UPR) are strongly activated in ALS. This activation correlates with increased expression of novel target genes, particularly those involved in ERAD such as HERPUD1 and SEL1L. Notably, SEL1L exhibits substantial upregulation in both PD and ALS, suggesting potential shared mechanisms [65]. Furthermore, reports indicate that ALS development is associated with dysregulation of stress granules (SGs). Wenbo Shi et al. reported that the HRD1-SEL1L ubiquitin ligase complex cooperatively maintains SG homeostasis in a stressor-dependent manner within the ubiquitin-proteasome system (UPS) and UPR. This occurs through an ATPase p97 and PERK-eIF2α signaling pathway that induces and mediates SG homeostasis to coordinate the UPR [66], indicating a specific role for SEL1L in the pathogenesis of ALS.

## SEL1L and tumors

Tumors are neoplasms formed by abnormal proliferation of cells within the body, often manifesting as localized masses of abnormal tissue (lumps). Tumor formation results from severe disruption of cellular growth regulation under the influence of various oncogenic factors. Benign and malignant tumors exhibit significant differences in cellular differentiation, growth patterns, metastatic capacity, and recurrence risk.

In recent years, the role of the ER in tumorigenesis and progression has garnered extensive attention. The ER participates in multiple biological processes including protein synthesis, folding, and transport. Research indicates that misfolding of key proteins within tumors may lead to the loss of tumor suppressor function [67]. The ER quality control system, particularly the unfolded protein response (UPR) and ER-associated degradation (ERAD) mechanisms, plays a complex role in cancer initiation and progression. It can either promote tumor development or potentially inhibit its growth, with the specific effect depending on the cellular microenvironment [68].

As an evolutionarily conserved component of ERAD, the SEL1L–HRD1 complex plays a vital role in the recognition and clearance of misfolded proteins. Altered expression of SEL1L and HRD1, either individually or in combination, is strongly associated with various human tumors [16]. ER quality-control pathways, including ERAD, may be activated in tumors to cope with the increased proteostatic burden associated with malignant proliferation [16, 68]. Deepening our understanding of ER quality control mechanisms like ERAD holds significant implications for developing novel tumor treatment strategies.

Under stress conditions such as hypoxia, nutrient deprivation, or increased protein misfolding, accumulation of unfolded proteins in the ER may trigger ERS, thereby activating the UPR. In tumor cells, the UPR enables cellular adaptation to adverse microenvironments like hypoxia and starvation, promoting survival and proliferation. However, if the UPR persists without effectively resolving ERS, it may induce apoptosis. As a key molecule maintaining ER function, SEL1L ensures proper protein folding and ER homeostasis by participating in processes like ERAD. Consequently, SEL1L is recognized as a critical factor associated with cell fate determination and tumor progression, particularly in malignant tumors.

### The role of SEL1L in tumors

In normal adult tissues, SEL1L expression is generally low, with relatively high expression observed in pancreatic cells [69]. However, in most tumor cells, SEL1L expression exhibits tissue specificity: it is downregulated in breast cancer, pancreatic cancer, and gastric cancer, while showing high expression in malignant gliomas [4, 69, 70]. In hepatocellular carcinoma, hepatocyte-specific loss of SEL1L or HRD1 promotes WNT5A misfolding, hepatocyte hyperproliferation, and liver tumorigenesis in mice, whereas higher SEL1L–HRD1 expression is associated with more favorable survival in the analyzed clinical cohort [16]. In breast, lung, esophageal, and prostate cancers, SEL1L protein levels increase during the transition from benign lesions to malignant tumors [67]. Immunohistochemical analysis further indicates that SEL1L is persistently expressed during the early carcinogenesis of esophageal, prostate, and non-small cell lung cancers, suggesting its potential as a biomarker for cellular malignant transformation [67]. In pancreatic and gastric carcinomas, SEL1L expression was reported to be reduced or absent in a tumor-type-specific manner [69]. In some early stages of tumor progression, SEL1L upregulation may represent a compensatory mechanism. However, its downregulation in poor-prognosis breast (and other) tumors indicates bidirectional regulatory roles in tumor progression.

#### SEL1L and liver tumors

Research indicates that SEL1L exerts an inhibitory effect in liver tumors, promoting the suppression of abnormal hepatocyte proliferation. Through long-term high-fat diet (HFD) feeding experiments, chronic carbon tetrachloride (CCl4) exposure models, and acute gene knockout models, it was discovered that SEL1L deficiency leads to abnormal accumulation of Wnt5a within hepatocytes, forming high-molecular-weight aggregates. In DEN-HFD-induced hepatocellular carcinoma models, SEL1L deficiency significantly increased liver tumor burden, suggesting that SEL1L deficiency promotes hepatocyte proliferation and hepatocellular carcinoma development [16]. Analysis of clinical samples revealed that high expression of SEL1L and HRD1 correlates positively with favorable prognosis in hepatocellular carcinoma patients, further supporting the critical role of SEL1L-HRD1 ERAD in inhibiting hepatocyte proliferation and tumorigenesis. At the clinical level, SEL1L and HRD1 protein levels were increased in hepatocellular carcinoma tissues, although SEL1L mRNA levels did not differ significantly between tumor and adjacent non-tumor tissues [16]. In HepG2 cells, genetic ablation of SEL1L induced mitochondrial fragmentation and dysfunction and altered transcriptional programs associated with cellular metabolism and tumor-related phenotypes [71]. Together with evidence from hepatocyte-specific mouse models showing that SEL1L–HRD1 deficiency promotes hepatocyte hyperproliferation and liver tumorigenesis through WNT5A dysregulation [16], these findings support a role for SEL1L–HRD1 in maintaining hepatic proteostasis and suppressing liver tumor development.

#### SEL1L in pancreatic cancer

Pancreatic cancer was among the first malignancies in which SEL1L was implicated. In a study of 74 primary pancreatic carcinomas, SEL1L expression was absent in 36% of tumors and was concordant with DPC4/SMAD4 expression in 69% of cases. SEL1L overexpression reduced clonogenicity, anchorage-independent growth, and xenograft tumor growth, suggesting a tumor-suppressive role in experimental pancreatic cancer models [72]. In pancreatic cancer cells, SEL1L overexpression reduced invasive behavior and promoted G1-phase accumulation, accompanied by modulation of PTEN and the matrix metalloproteinase inhibitors TIMP1 and TIMP2 [73]. In a cohort of 497 Caucasian patients with pancreatic ductal adenocarcinoma, the SEL1L rs12435998 variant was associated with age at diagnosis and survival in selected patient subgroups, supporting its potential prognostic relevance [74]. However, the clinical and mechanistic significance of SEL1L in pancreatic cancer requires further validation.

### SEL1L protein variants and tumors

Researchers identified two novel endogenous truncated SEL1L protein variants (p38 and p28) in multiple cell lines and analyzed their expression and distribution in breast cancer cell lines SKBr3 and MCF7, multiple myeloma cell line KMS11, and non-tumor cell lines MCF10A and 293FT. Results showed p38 was expressed in all tested cell lines with higher levels in cancer cells, while p28 was detected only in the highly malignant metastatic breast cancer cell line SKBr3 [75]. Through SDS-PAGE, immunoblotting, and ERS/UPR induction models, it was found that p38 and p28 exhibit secretory activity under stress conditions. Their biochemical properties and subcellular localization differ from full-length SEL1L, with enrichment primarily in late Golgi membrane vesicles and partial release into the extracellular space [75]. Further biochemical analysis revealed that the isoelectric points of p38 and p28 range between 5.25 and 5.50, slightly lower than that of full-length SEL1L (approximately 5.8), supporting the theory of their specific compartmental localization. Although p38 is difficult to recover in immunoprecipitation, its antigenicity remains detectable in immunohistochemistry, suggesting that its native conformation may influence antibody recognition. This study expands our understanding of the functional roles of SEL1L variants in ERS/UPR responses and lays the groundwork for exploring their potential involvement in tumorigenesis and progression.

## SEL1L and metabolic diseases

As the basic structural and functional units of the human body, cells carry out essential processes including metabolism, cellular communication, protein synthesis, and modification. SEL1L is a highly conserved ER transmembrane protein and a core component of the ERAD pathway, playing a crucial role in cellular metabolic activities. ERAD is a major protein quality control mechanism responsible for the disposal of misfolded proteins. The SEL1L–HRD1 complex represents one of the most evolutionarily conserved Hrd1-centered branches of ERAD, with functional roots in the yeast Hrd3p–Hrd1p complex. This evolutionary continuity is consistent with its fundamental role in maintaining ER proteostasis across diverse cell types, including adipocytes, pancreatic β-cells, neurons, hepatocytes, and immune cells [76]. Consequently, it is intricately linked to lipid metabolism, diabetes, renal metabolic diseases, and hepatic metabolic disorders, as detailed in Table 1.

**TABLE 1:** SEL1L in metabolic diseases.

Metabolic disease	Mechanism of action	Key target	Common feature
Lipid metabolism disorders	SEL1L promotes lipoprotein lipase (LPL) secretion from the endoplasmic reticulum (ER) and thereby contributes to systemic lipid homeostasis; Sel1l deficiency causes ER retention of LPL and impairs lipid metabolism [14]	Lipoprotein lipase (LPL)	Across these tissues, SEL1L–HRD1 ERAD links ER protein quality control to tissue-specific substrate handling and metabolic homeostasis, with disease phenotypes determined by the relevant substrate and cell type
Diabetes-related β-cell dysfunction	β-cell-specific Sel1l deficiency causes loss of β-cell identity and reduced insulin content through dysregulated TGF-β signaling; SEL1L–HRD1 ERAD promotes degradation of TGF-β receptor 1 [77]	TGF-β receptor 1 (TGFBR1)	
Congenital nephrotic syndrome (CNS)-Like phenotype	The SEL1L–HRD1 ERAD complex promotes nephrin maturation and proteostasis in podocytes; podocyte-specific Sel1l deficiency causes nephrin misprocessing, glomerular filtration defects, and a CNS-like phenotype [78]	Nephrin (NPHS1)	
Hepatic metabolic disorders	In hepatocytes, SEL1L–HRD1 ERAD regulates FGF21 levels through CREBH and controls systemic metabolism; it also maintains systemic iron homeostasis by regulating ceruloplasmin turnover [15, 79]	FGF21/CREBH; ceruloplasmin	

### SEL1L and lipid metabolism disorders

Lipids primarily function as an oxidative energy source in the body. Lipid metabolism is regulated by genetic, neurohumoral, hormonal, enzymatic, and hepatic factors. Abnormalities in these factors can lead to lipid metabolism disorders and related pathophysiological changes, such as hyperlipidemia, obesity, and fatty liver disease. Recent research over the past decade has identified connections between lipid metabolism disorders and the SEL1L molecule. The central role of SEL1L in lipid metabolism is exemplified by its regulation of lipoprotein lipase (LPL) [80]. LPL is a key lipolytic enzyme acting on circulating triglycerides, promoting their uptake and utilization in adipose tissue to regulate systemic lipid distribution and metabolism [81]. The maturation of LPL protein requires stabilization by the lipase maturation factor 1 (LMF1), an ER-resident protein that promotes LPL dimerization in the ER. Studies by Sha et al. indicate that SEL1L facilitates the transport of LPL from the ER to the Golgi and its subsequent secretion by forming a stable complex with LPL and LMF1 [14], thereby indirectly participating in systemic lipid metabolism. Under conditions of SEL1L deficiency, LPL fails to be efficiently released from the ER, leading to its retention and aggregation within the organelle, which subsequently causes lipid metabolism dysfunction. Abnormal fat accumulation in hepatocytes can lead to non-alcoholic fatty liver disease, potentially linked to LPL retention in the ER due to SEL1L deficiency. Furthermore, SEL1L influences fatty acid synthesis and oxidation by regulating the expression of lipid metabolism-related genes, thereby playing a role in the development and progression of fatty liver disease.

### SEL1L and diabetes

Diabetes mellitus is a chronic disease characterized by hyperglycemia resulting from absolute or relative insulin deficiency and impaired insulin utilization. Pancreatic β-cells are the sole cell type capable of secreting insulin, and their dysfunction is a primary cause of diabetes. Research has shown that SEL1L is abundantly expressed in pancreatic β-cells and plays a crucial role in maintaining ER homeostasis and ensuring the correct folding of proteins involved in insulin secretion, thereby supporting normal insulin release. Studies by Shrestha et al. demonstrate that SEL1L deficiency disrupts ER homeostasis in pancreatic β-cells, leading to accumulation of misfolded proteins, ER stress, apoptosis, and impaired insulin synthesis and secretion, ultimately contributing to diabetes [77]. This process not only damages β-cell structure and function but can also lead to loss of β-cell identity. SEL1L primarily functions by forming a complex with the E3 ubiquitin ligase HRD1, which then operates within the ERAD pathway. Research by Shrestha N et al. indicates that deficiency of the SEL1L-HRD1 protein complex or autophagy in pancreatic β-cells can trigger diabetes through distinct mechanisms: β-cell dedifferentiation or death [82].

These findings highlight the significant role of the SEL1L molecule in the pathogenesis of diabetes, although the detailed molecular mechanisms of how the SEL1L-HRD1 complex acts on pancreatic β-cells require further exploration. Nevertheless, they suggest a new therapeutic direction for diabetes: interventions targeting SEL1L. Restoring SEL1L function could improve ER homeostasis in pancreatic β-cells, reduce misfolded protein accumulation, inhibit ER stress and apoptosis, protect pancreatic β-cell function, promote normal insulin secretion, and help control blood glucose levels.

### SEL1L and renal diseases

Proteinuria, congenital nephrotic syndrome (CNS), and renal tubulointerstitial fibrosis are all conditions involving renal dysfunction. The glomerular filtration barrier is a key structure for maintaining internal homeostasis, and its damage often leads to proteinuria. SEL1L-HRD1 ERAD exhibits cell-type-specific expression in the kidney, with abundant expression localized to glomerular podocytes, which are uniquely responsible for maintaining filtration barrier integrity [78]. It helps maintain the integrity of the glomerular filtration barrier by regulating podocyte differentiation, proliferation, and apoptosis. Congenital nephrotic syndrome (CNS) is a common pediatric kidney disease characterized by increased permeability of the glomerular basement membrane and massive urinary protein loss. Nephrin, a membrane protein associated with CNS, is also a substrate for ERAD. Nephrin is a protein synthesized and matured in the ER; if misfolded, it can be degraded via the ERAD pathway. Newly synthesized nephrin is prone to misfolding and can be ubiquitinated by the SEL1L-HRD1 complex for proteasomal degradation. Research by Yoshida S et al. suggests that in the absence of functional ERAD, misfolded nephrin accumulates in the ER, potentially undergoing refolding or interfering with the normal maturation of nascent nephrin [78], thereby contributing to CNS. Concurrently, SEL1L deficiency increases ER stress in podocytes and impairs cytoskeletal remodeling, which often manifests as proteinuria in CNS. Renal tubulointerstitial fibrosis is a major pathological process in the progression of chronic kidney disease to end-stage renal disease. Distinct from its function in podocytes, SEL1L in renal tubular epithelial cells acts as an intrinsic regulator of collagen biosynthesis and turnover; it directly mediates the clearance of extracellular collagen [83]. SEL1L mitigates the degree of tubulointerstitial fibrosis and protects renal function by inhibiting ER stress and apoptosis in renal tubular epithelial cells. SEL1L deficiency leads to pathological accumulation of collagen within renal tubular epithelial tissue, contributing to tubulointerstitial fibrosis.

### SEL1L and hepatic diseases

Common liver diseases such as hepatitis and cirrhosis are closely related to hepatocyte damage and regeneration. Research indicates that hepatocyte injury and regeneration involve multiple molecular mechanisms, including the Notch and Hippo signaling pathways. Recent studies show that normal hepatocyte physiology is also closely linked to the SEL1L molecule. Its role in liver diseases is mainly manifested in two aspects: (1) Protecting the liver from damage by regulating ER stress and inflammatory responses within hepatocytes. Studies by Bhattacharya A et al. found significantly upregulated expression of ER stress markers (such as Bip and Chop) and concurrent release of inflammatory factors in the livers of SEL1L-deficient mice, indicating that SEL1L deficiency exacerbates hepatic ER stress and inflammatory responses, leading to liver injury. (2) Maintaining hepatic iron metabolic balance by regulating the stability of iron metabolism-related proteins. Research by Thepsuwan P et al. indicates that SEL1L also influences hepatic iron homeostasis by regulating the stability of proteins like ceruloplasmin (CP) [79].

Notably, SEL1L exerts divergent, tissue-specific effects across kidney and liver. In the kidney, the primary substrates of SEL1L-HRD1 ERAD are nephrin and collagen, supporting filtration barrier architecture and extracellular matrix homeostasis; in the liver, SEL1L predominantly regulates inflammatory signaling and ceruloplasmin-dependent iron metabolism. Such organ-specific outcomes arise from differences in cell-type expression profiles, available ERAD substrates, and downstream signaling networks between podocytes, renal tubular epithelial cells and hepatocytes.

In fact, SEL1L-HRD1 ERAD plays a pivotal role in normal physiological activities such as food intake regulation, energy metabolism, and water homeostasis. It governs the degradation of misfolded proteins (ERAD). Many physiological processes, including glucose and lipid metabolism, require enzyme catalysis, and enzymes are themselves proteins. When SEL1L is deficient and ERAD is impaired, the accumulating misfolded proteins in the ER can disrupt normal metabolic activities, leading to the diseases described above. Future research should further elucidate the specific mechanisms of SEL1L in these diseases and develop targeted therapies against SEL1L, offering new strategic approaches for treating related conditions.

## SEL1L plays a crucial role in the immune system

SEL1L, as a key component in the ERAD pathway, plays multiple roles in the immune system. Beyond participating in the degradation of misfolded or unfolded proteins within the ER, SEL1L also finely regulates the development, function, and immune responses of immune cells at multiple levels.

### The role of SEL1L in innate immunity

Research indicates that the SEL1L–HRD1 complex plays a crucial role in innate immunity, particularly within the STING (Stimulator of Interferon Genes) signaling pathway. STING is an innate immune signaling protein localized to the ER. Upon responding to the second messenger cGAMP generated after cytoplasmic DNA is sensed by cGAS, STING undergoes a conformational change. STING then recruits and activates the kinase TBK1, which phosphorylates interferon regulatory factor 3 (IRF-3), thereby inducing the expression of key inflammatory cytokine genes involved in innate immunity, such as type I interferon (IFN) [84]. However, excessive STING signaling may lead to overactivation of inflammatory mediators and tissue damage, contributing to various immune disorders including STING-associated vascular disorders (SAVI), Aicardi-Goutières syndrome (AGS), and systemic lupus erythematosus (SLE) [85]. Thus, strict regulation of STING activity is essential for maintaining immune homeostasis.

SEL1L-HRD1 ERAD regulates cGAS/STING signaling via STING. In its inactive state, the SEL1L–HRD1 complex continuously recognizes and ubiquitinates newly synthesized STING protein, degrading it through the proteasome pathway. This limits the size of the pool of activatable STING, thereby negatively regulating the STING signaling pathway [5]. In macrophages, deletion of either SEL1L or HRD1 leads to significantly elevated STING protein levels, thereby enhancing STING signaling and cytokine production. This demonstrates the critical role of SEL1L–HRD1 in maintaining STING signaling homeostasis [5].

### The role of SEL1L in immune cell function

#### Impact on T cell survival, development, and function

The role of SEL1L in adaptive immunity is primarily manifested in T cell survival, development, and function. By finely regulating the PERK signaling pathway and the IL-15 receptor-mediated mTORC1 axis, SEL1L plays a crucial role in maintaining CD8^+^ T cell survival and homeostasis. Studies indicate that SEL1L deficiency leads to excessive activation of the PERK-ATF4-CHOP-Bim signaling pathway, resulting in significantly reduced survival capacity of CD8^+^ T cells both *in vivo* and *in vitro*, manifested as increased apoptosis [86]. SEL1L deficiency increases IL-15 receptor expression and signaling in CD8^+^ T cells, thereby enhancing mTORC1–c-MYC activity and triggering metabolic reprogramming that compromises CD8^+^ T-cell survival and homeostasis [86]. Consistent with these phenotypic observations, loss of Sel1L impairs CD8^+^ T-cell effector function and memory formation following acute viral infection [87].

SEL1L is essential for T cell thymic development, particularly at the β-selection checkpoint. In mice, T-cell-specific Sel1l deletion impairs thymocyte β-selection and αβ T-cell development, resulting in the accumulation of CD4^−^CD8^−^ double-negative stage 3 (DN3) thymocytes and a reduced CD4^+^CD8^+^ double-positive (DP) thymocyte compartment [88].

Regarding T cell function, activated CD8^+^ T cells undergo dynamic ER stress, during which SEL1L is significantly upregulated and plays a crucial role in maintaining T cell energy and cytokine production [87]. SEL1L deficiency leads to impaired CD8^+^ T cell function, including reduced effector molecule production and impaired memory formation [87]. This demonstrates the critical role of SEL1L in regulating T cell responses and ensuring the formation of immune memory.

#### Regulation of B-cell receptor signaling

Research has revealed that SEL1L also participates in regulating B-cell receptor (BCR) signaling. Research indicates that in STING V154M knock-in mouse models, STING interacts with the SEL1L-HRD1 complex. This mechanism leads to rapid degradation of the BCR, revealing a novel role for SEL1L in regulating BCR stability and signaling [89]. This may impact B cell development, activation, and antibody production.

### The role of SEL1L in immune-related diseases

#### SEL1L and autoimmune diseases

The critical role of SEL1L in maintaining immune homeostasis is also reflected in its impact on autoimmune diseases. Experimental autoimmune encephalomyelitis (EAE) serves as a widely used animal model for multiple sclerosis. Studies reveal that T cell-specific SEL1L deficiency exacerbates EAE severity, manifested by higher clinical scores and more pronounced central nervous system inflammation. Further analysis indicates that SEL1L deficiency promotes Th1 and Th17 cell differentiation, potentially contributing to the intensified EAE [90]. This demonstrates SEL1L’s crucial role in regulating T cell subset balance and preventing excessive activation of autoimmune responses.

#### SEL1L and infectious diseases

Research indicates that SEL1L may play a crucial role in viral infection processes. For instance, in hepatitis B virus (HBV) infection, SEL1L expression levels negatively correlate with viral load, and SEL1L regulates HBV replication through the ERAD pathway [91].

It is noteworthy that ER proteostasis pathways may influence viral infections, particularly those involving positive-strand RNA viruses that replicate on ER-derived membranes. In plant virus infections, Sec62-dependent, UPR-associated ER-phagy restricts infection by ER-replicating positive-strand RNA viruses [92]. In contrast, during flavivirus infection, a Derlin-2/SEL1L-dependent ERAD pathway selectively degrades excess membrane-anchored viral nonstructural proteins, thereby maintaining viral protein homeostasis and supporting viral genome replication [93]. As a core adaptor protein of the ERAD pathway, SEL1L may influence viral replication efficiency and infectivity by regulating the degradation homeostasis of viral proteins within the ER. During flavivirus infection, the Derlin-2–SEL1L–HRD1 ERAD complex mediates the degradation of accumulated viral membrane nonstructural proteins to maintain viral protein homeostasis, which is essential for flavivirus propagation [93].

Furthermore, SEL1L mediates complex regulatory crosstalk across multiple branches of the UPR. Upon chronic ER stress, SEL1L may govern cell fate decisions between adaptive survival and apoptotic initiation, likely by modulating signaling through the PERK, IRE1, and ATF6 pathways [66, 94]. This regulatory balance is especially pertinent to viral infections: persistent ER stress triggered by viruses can either mount host protective responses or be exploited to support viral propagation. The exact mechanisms whereby SEL1L fine-tunes this equilibrium, as well as its tissue-specific functions (e.g., in the liver), remain to be fully elucidated. Accordingly, systematic dissection of the divergent and convergent functions of SEL1L during diverse RNA virus infections will deepen our understanding of host–virus interplay and may uncover new avenues for developing broad-spectrum antiviral therapeutics.

#### The role of SEL1L in tumor immunity

SEL1L–HRD1 may link tumor biology with immune regulation. In hepatocellular carcinoma, higher SEL1L and HRD1 expression levels are associated with better overall survival, whereas hepatocyte-specific disruption of SEL1L–HRD1 promotes hepatocyte hyperproliferation and liver tumorigenesis [16]. In Huh7 hepatocellular carcinoma cells, pharmacological or genetic inhibition of SEL1L-mediated ERAD suppresses cell proliferation and migration and promotes apoptosis [95]. In addition, the SEL1L–HRD1 complex restrains basal STING-mediated innate immune signaling by promoting the degradation of nascent STING [5]. Together, these findings suggest that SEL1L–HRD1 coordinates ER proteostasis with tumor suppression and innate immune regulation.

## Discussion

In recent years, research on related pathways has garnered increasing attention from scholars. ERAD plays a crucial and extensive role in maintaining normal metabolic processes. As a key molecule in the ERAD process, SEL1L influences various aspects of the body through multiple pathways across numerous domains—from glycolipid metabolism and immune regulation to tumor development and protein deposition in the nervous system. Current research increasingly links SEL1L to neurological disorders. With the discovery of new pathways and factors, targeted therapeutic approaches focusing on SEL1L hold promise for future applications.

## Data Availability

The original contributions presented in the study are included in the article/supplementary material, further inquiries can be directed to the corresponding author.
